# Effective Therapy Against Severe Anxiety Caused by Cancer: A Case Report and Review of the Literature

**DOI:** 10.7759/cureus.8414

**Published:** 2020-06-02

**Authors:** Ryo Sakamoto, Atsuko Koyama

**Affiliations:** 1 Psychosomatic Medicine, Kindai University Faculty of Medicine, Osakasayama City, JPN

**Keywords:** rare cancer, adenoid cystic carcinoma, anxiety, restlessness, quetiapine

## Abstract

Anxiety can make it difficult for patients to manage their illness. Therefore, it is important to reduce their anxiety if possible. However, few studies have examined the efficacy of drugs in the treatment of anxiety in patients with cancer. Our case had failed to respond to benzodiazepines, and it was difficult to use a selective serotonin reuptake inhibitor (SSRI) as the next drug. This case report describes the effective use of quetiapine to treat anxiety. We report this rare case along with a literature review. Few studies have assessed the treatment of anxiety in patients with rare cancers. In our case, quetiapine effectively alleviated anxiety associated with cystic adenoid carcinoma. However, in clinical practice, it is possible that anxiety is treated without differentiating the effects of cancer status, e.g. life prognosis, treatment progress. In our patient, benzodiazepines had no effect on anxiety. Thus, different drugs may be required to treat anxiety associated with cancer. The present study demonstrated that quetiapine is a useful modality for the palliative care of patients with rare cancer and intractable anxiety. Quetiapine may be an effective alternative to benzodiazepines (BZ) and SSRIs for treating anxiety in patients with cancer. However, further investigation is needed to clarify the efficacy of treatments for anxiety associated with rare cancers.

## Introduction

Anxiety is a common problem in patients with cancer. The prevalence of anxiety in patients with cancer is 10%-13% [1, 2]. Anxiety can make it difficult for patients with cancer to manage their illness. Therefore, it is important to reduce their anxiety whenever possible. Unfortunately, the treatment of anxiety in patients with cancer has not been extensively studied. Anxiolytic drugs and selective serotonin reuptake inhibitors (SSRIs) are prescribed as standard treatments for anxiety in patients who do not have cancer [3]. Meanwhile, approximately 70% of patients with anxiety related to cancer are prescribed benzodiazepines [2]. However, few studies have examined the efficacy of drugs in reducing anxiety in patients with cancer [4, 5]. Therefore, we performed a literature review of the treatment of anxiety in patients with cancer. In addition, we report a case of severe anxiety associated with a rare cancer. Patients diagnosed with rare cancers report more severe anxiety than the general population of patients with cancer [6]. Our case did not respond to benzodiazepines, and it was difficult to use an SSRI as the next drug. In this case, quetiapine was proven effective against anxiety. We report this rare case along with a literature review.

## Case presentation

Our patient, a 33-year-old woman, presented to our hospital with a right lower abdominal mass 13 months previously. Two months later, the mass was surgically removed. Examination secured a diagnosis of stage I adenoid cystic carcinoma. One month later, the patient visited a medical oncology department for consultation regarding their future treatment plan. Radiological examination revealed no cancer metastasis. However, the doctors were not able to obtain sufficient information about the possible future progression of the malignancy, which increased the patient’s anxiety. The patient did not have any mental illnesses such as depression and schizophrenia, nor did she have any history of such illnesses. As her anxiety did not improve, she was prescribed lorazepam at an oral daily dose of 1.5 mg. However, her anxiety did not improve, and she began to experience sleep disorders, which seriously affected her subjective quality of life and overall daily function. One month later, she presented to our outpatient clinic for consultation concerning her psychological symptoms. At the first visit, she was tearful, stating “I’m not sure what to do. I understand that I don’t know much about adenoid cystic carcinoma, but I keep getting anxious.” She had lost 6 kg over the prior four weeks owing to anxiety-related appetite loss. She worried excessively about her future, and had fallen into a negative thinking cycle owing to a fear of death. Her immediate goal was to complete a medical examination to obtain a second opinion about her adenoid cystic carcinoma from a specialty hospital, hoping that expert medical advice would relieve her anxiety. However, because her anxiety affected her daily life, she was unable to travel far to visit a hospital. Because lorazepam had no effect, we decided to taper off this drug. We wanted to use an SSRI as the next treatment, but this drug class can possibly induce serotonin syndrome. Therefore, we prescribed 12.5 mg of quetiapine orally as required. To reduce the adverse effects of quetiapine (e.g., cerebrovascular events, neuroleptic malignant syndrome, metabolic changes, suicidal thoughts and behaviors), we titrated the dose down to a slightly safer amount. On a return visit seven days later, the patient was prescribed quetiapine three times a week. At this dosage, her sleep increased to approximately 6 h per night, and she had no obvious side effects. However, she reported that “Focusing on my housework or parenting helps keep my mind off adenoid cystic carcinoma. Out of nowhere, I’ll suddenly remember adenoid cystic carcinoma.” We told her that she did not necessarily need to stop thinking negatively to overcome her anxiety. On a return visit 14 days later, the patient said “I was so anxious at the thought of having to receive long-term medical examinations for 5 or 10 or more years. But I gradually began to think that at the moment I may be all right for 5 or 10 years.” Over the course of several consultations, she gradually became able to lead her daily life despite concerns about adenoid cystic carcinoma. The patient’s psychological symptoms stabilized to the point that she only required treatment with quetiapine a few times a month. Two months later, she obtained her desired second opinion, and she is currently being treated by that institution.

## Discussion

The prevalence of anxiety in patients with cancer is 10%-13% [1, 2]. Patients diagnosed with rare cancers report more severe anxiety than the general population of patients with cancer [6]. To investigate the ability of patients with cancer to manage the associated anxiety, we searched PubMed and MEDLINE using the terms cancer and medications (benzodiazepine or antidepressant or antipsychotic). Eight cases of patients with cancer who were treated for anxiety were identified. These cases are summarized in Table 1.

**Table 1:** Brief review of cases of anxiety to cancer F: female; GIST: Gastrointestinal stromal tumor; M: male.

No.	First author	Age/sex	Cancer	Psychotropic treatment (medications)	Psychotropic treatment (psychotherapy)	Clinical course and treatment
1	Sakamoto R. (this article)	33/F	adenoid cystic carcinoma	quetiapine	no	See text. A 33-year-old. The doctors were not able to obtain enough information about possible future progress, which increased the patient’s anxiety. She worried excessively about her future, and had fallen into a negative thinking cycle owing to a fear of death.
2	Miura M. [7]	11/M	acute lymphocytic leukemia	duloxetine	no	An 11-year-old. By treatment with medication, loneliness during isolation because of an immunocompromised state, anxiety about entering junior high school. After umbilical cord blood transplant Day 64, he was prescribed 10 mg of duloxetine. Furthermore, to address the patient’s mental anxiety, a meeting was held with the teacher whose class the patient was expected to attend. By those medications, anxiety improved.
3	Rodríguez-Mayoral O. [8]	39/F	cervical cancer	sertraline	no	A 39-year-old. Patient diagnosed with cervical cancer stage IVB, consulted to the emergency department after a suicide attempt by hanging. She reported intense pain, unresponsive to analgesics, and had a history of persistent suicidal ideation. Accordingly, antidepressant treatment was started (sertraline 50 mg/d). The depressive symptoms decreased.
4	Xu M. [9]	61/M	small cell carcinoma	duloxetine clonazepam	no	A 61-year-old. One month after chemoradiotherapy, the patient developed fever without obvious causes. The patient successively received intravenous infusions of antibiotics for anti-infection treatment in another hospital, but his temperature did not improve. Fear of the malignant tumor caused mental stress, leading to a series of pathophysiological changes that caused fever. He was prescribed 30 mg of duloxetine and 0.5 mg of clonazepam. Eventually, those medications were two times higher than first time. By those medications, anxiety symptoms improved.
5	John P. [10]	68/F	GIST and pancreatic cancer	paroxetine lorazepam	no	She had symptoms of anxiety on cycle 1 day 15 of gemcitabine. Thus, she reported difficulty sleeping which included waking up frequently through the night and therefore sleeping for two to three hours at a time. She informed us that she was worried about her cancer. She was started on paroxetine 10 mg po once daily as well as lorazepam 1 mg po 1-2 tablets every six hours as needed. However, approximately 2 weeks after initiation of paroxetine, she developed a chin tremor. Therefore, paroxetine was discontinued.
6	Montel S. [11]	60/F	breast cancer	bromazepam	cognitive–behavioural therapy (CBT)	A 60-year-old. Since the beginning of her cancer, and more especially since the end of her treatment, she felt that her anxiety increased and was more focused on the potential recurrence of cancer. She used to take bromazepam (anxiolytic) one-fourth daily and CBT training.
7	Jozuka H. [12]	52/F	hepatocellular carcinoma	fluvoxamine flutoprazepam lormetazoram	no	A 52-year-old. She worked as a beautician. Since her husband was a chronic gambler she was obliged to provide for the whole household and this situation caused her permanent stress. She was suffering from sleep disturbance, depressive mood and cancer phobia. Therefore, fluvoxamine (50 mg/day), flutoprazepam (2 mg/day) and lormetazoram (1 mg/day) were administered.
8	O'Hayer CVF. [13]	54/F	Pancreatic ductal adenocarcinoma	no	Acceptance and Commitment (ACT)	A 54-year-old. Throughout the treatment, she had flat effect, tearfulness. She declined the initiation of a selective serotonin reuptake inhibitor for treatment. However, she agreed to psychotherapy. She attended five weekly sessions of ACT. As a result of ACT, the patient was able to be present with emotions, and discuss with her previously estranged family and girlfriends.

To the best of our knowledge, this is the first reported use of quetiapine to reduce anxiety associated with adenoid cystic carcinoma. The most important point of this case is that quetiapine was effective against anxiety caused by a rare cancer. Benzodiazepines are used to manage several symptoms in patients with cancer, including anxiety, insomnia, confusion, and restlessness [14]. However, there are no reports that benzodiazepines are effective against adenoid cystic carcinoma-induced anxiety, and they had no effect in this case. Presumably, rare cancers cause anxiety that differs from that associated with common cancers, in line with previous findings that patients with rare cancers report more severe anxiety than the general population of patients with cancer [6]. In case SSRIs are ineffective, serotonin noradrenaline reuptake inhibitors (SNRIs) and other antidepressants may be add-on option. In addition to SSRIs, SNRIs are treatment drug against anxiety. On the other hand, as with SSRIs, the disadvantages need time to take effect. We had used quetiapine in anxiety with common cancers. We have a good impression of quetiapine in terms of early effect development time than SSRIs and SNRIs. In this case, anxiety was associated with sweating, trembling, abdominal distress, fear of dying, hot flashes, feeling keyed up and mental tension. These symptoms fulfilled the diagnostic criteria in generalized anxiety disorder. Quetiapine substantially reduces somatic fear symptoms [15]. It is possible that anxiety caused by rare cancers such as adenoid cystic carcinoma is associated with somatic fear symptoms. There is evidence that generalized anxiety disorder is a concurrent predictor of fear symptoms [16]. It has been suggested that quetiapine is effective for treating generalized anxiety disorder [17]. The fact that rare cancers are likely to be associated with fear symptoms and generalized anxiety may explain why quetiapine was more effective than benzodiazepines in this case. Although this drug has a short half-life and is easy to use, there are concerns regarding its frequent off-label use. There are also concerns about the abuse of quetiapine [18]. Therefore, physicians should use caution when prescribing quetiapine to relieve anxiety.

Miura et al. and Rodriguez-Mayoral et al. reported cases of patients who received antidepressants alone to treat cancer-related anxiety [7, 8]. The most important point of these cases is that for relatively young patients, antidepressants alone appear to be effective against anxiety caused by cancer. The percentage of patients who used benzodiazepines increased with age [19]. Thus, older patients may require benzodiazepines for anxiety. In fact, four cases older than 50 years identified in our literature review received benzodiazepines alone or in combination with SSRIs to treat anxiety [9-12].

Two cases received psychotherapy to treat anxiety associated with cancer. O’Hayer et al. reported that a patient with pancreatic ductal adenocarcinoma declined to start SSRI therapy even though pancreatic ductal adenocarcinoma is associated with significantly higher rates of depression and anxiety than stage- and prognosis-matched advanced tumors of other origins [13, 20]. The case reported by Montel was treated with benzodiazepines because this patient exhibited generalized anxiety disorder [11]. It is possible that these were associated with severe anxiety, and psychotherapy (e.g., ACT, CBT ) would be effective in these cases.

There are two unique features of our case in comparison with the other reviewed cases. First, few studies have examined the treatment of anxiety associated with rare cancers. In our case, quetiapine was effective for treating anxiety associated with cystic adenoid carcinoma. However, in clinical practice, it is possible that anxiety is treated without differentiation by cancer status, e.g., life prognosis, treatment progress. Benzodiazepines had no effect against anxiety in our case. Thus, the efficacy of drugs against cancer-associated anxiety may differ by cancer type. In the future, it will be necessary to consider the cancer status when selecting treatments for cancer-associated anxiety. Second, our patient did not receive SSRIs. We selected quetiapine in this case because SSRIs can increase the risk of serotonin syndrome.

In some cases, patients with more severe anxiety can exhibit impatience. Our patient desired a rapid effect because of a significant decrease in activities of daily living, and she had difficulty completing an outpatient visit for anxiety. SSRIs require approximately one month for efficacy to become apparent. Therefore, the use of SSRIs would have been difficult in this case. Consequently, antipsychotics may represent a treatment option for benzodiazepine-refractory anxiety. Given the potential efficacy of quetiapine, we believe it would be suitable to use this drug for this indication.

## Conclusions

The present study demonstrated that quetiapine is a useful modality for the palliative care of patients with rare cancer and intractable anxiety. Quetiapine may be more effective than benzodiazepines and SSRIs for treating anxiety associated with cancer. Accordingly, it has been identified as a potential future treatment option. In addition, psychotherapy (ACT, CBT) is also suggested to be effective in the treatment of severe anxiety in patients with cancer. However, further studies are needed to identify the optimal treatment strategies for anxiety in patients with rare cancers.
